# Iatraleptic Treatment of Dropsy

**Published:** 1829-01-01

**Authors:** 


### IATRALEPTIC TREATMENT OF DROPSY. By Dr. GUIBERT.

Under the above head Dr. Guibert has published a memoir, in which he develops a modification of treatment not usually employed in the cure of dropsical effusions. The author properly observes that dropsy is rarely an idiopathic disease, being generally dependent on inflammation, acute or chronic, of the peritoneum or of the pleura—or on some organic disease of the viscera contained in the chest or abdomen. But whatever may have been the *cause*, we are called upon to remove the *effect*—the effusion, which, in itself, becomes a formidable and even fatal disease. Wide experience has demonstrated that diuretics are our main resources for the removal of the effusion.

---

Purgation, salivation, and increased secretions from the various glands, have occasionally succeeded; yet they are precarious, and sometimes exhausting remedies. But diuretics themselves are often very irritating to the stomach and bowels; and if we could introduce them into the system through the medium of the skin, we should certainly make an improvement in the treatment of dropsy. This improvement, then, is the object of Dr. Guibert. The following is the formula of his diuretic liniment:—

℞. Tinct. scillæ,
——— digitalis,
——— Sem. colchici, āā ℥ss.
Olei camphorat. et ammoniat. ℥iss.
Misce ft. linimentum.

With this liniment the chest, the abdomen, or the members affected with dropsy, are to be well rubbed twice or thrice a day, by means of a piece of flannel. Dr. G. also prescribes internally the following pills:—

℞. Extr. lactucæ, ʒj.

Pulv. scillæ.

——— digitalis.

Nitrat. potassæ, āā ℈ij.

Oxymellis q. s. ut fiant pil. 72, quarum capiat æger j. mane ac vesperi, augente dosi de die in diem.

Several cases of hydrothorax, ascites, and anasarca are related, as successfully treated by the foregoing remedies. But these cases need not be detailed. The increase of the urinary secretion very obviously followed the external and internal employment of the diuretics, and the decrease of the dropsical effusions was a natural consequence. We would advise a trial of the diuretic liniment. As for the pills, a better form perhaps might be struck out by the English prescriber.—REV. MED.

---

